# Mesenchymal Stem Cell Therapy for Osteoarthritis: The Critical Role of the Cell Secretome

**DOI:** 10.3389/fbioe.2019.00009

**Published:** 2019-01-29

**Authors:** Patrizio Mancuso, Swarna Raman, Aoife Glynn, Frank Barry, J. Mary Murphy

**Affiliations:** ^1^Regenerative Medicine Institute (REMEDI), Biosciences, National University of Ireland Galway, Galway, Ireland; ^2^Centre for Research in Medical Devices (CÚRAM), Biosciences, National University of Ireland Galway, Galway, Ireland

**Keywords:** osteoarthristis, mesenchymal stem cells, immunomodulation, secretome, paracrine action, chondroprotection

## Abstract

Osteoarthritis (OA) is an inflammatory condition still lacking effective treatments. Mesenchymal stem/stromal cells (MSCs) have been successfully employed in pre-clinical models aiming to resurface the degenerated cartilage. In early-phase clinical trials, intra-articular (IA) administration of MSCs leads to pain reduction and cartilage protection or healing. However, the consistent lack of engraftment indicates that the observed effect is delivered through a “hit-and-run” mechanism, by a temporal release of paracrine molecules. MSCs express a variety of chemokines and cytokines that aid in repair of degraded tissue, restoration of normal tissue metabolism and, most importantly, counteracting inflammation. Secretion of therapeutic factors is increased upon licensing by inflammatory signals or apoptosis, induced by the host immune system. Trophic effectors are released as soluble molecules or carried by extracellular vesicles (ECVs). This review provides an overview of the functions and mechanisms of MSC-secreted molecules found to be upregulated in models of OA, whether using *in vitro* or *in vivo* models.

## Mesenchymal Stromal Cells

Since described by Friedenstein (Friedenstein et al., 1966), mesenchymal stem/stromal cells (MSCs) have been the focus of research efforts to exploit their therapeutic potential. Due to their immune-evasive nature, MSCs release immunomodulatory factors which allow them to escape rejection mechanisms for sufficient time to exert their therapeutic action (Ankrum et al., 2014). MSCs also express a variety of cytokine and chemokine receptors, such as CXCR4, CXCR7, and CCR7, enabling migration to sites of injury and inflammation (Sasaki et al., 2008; Liu et al., 2012).

As a paradigm for tissue regeneration, MSCs have been used for many orthopedic conditions, including osteoarthritis (OA). The first successful treatment used a caprine model of OA involving anterior cruciate ligament transection combined with total medial meniscectomy (Murphy et al., 2003). Direct intra-articular (IA) delivery of autologous bone marrow (BM)-MSCs, 6 weeks after injury, led to meniscal repair and chondro-protection. The green fluorescent protein (GFP)-transduced cells were detectable in the synovial capsule, fat pad and newly-formed meniscus, but not in articular cartilage. This work led to the hypothesis that MSCs act via alternate mechanisms to cell replacement i.e., trophic mechanisms to promote tissue regeneration through modulation of the host environment and/or stimulation of endogenous progenitors (Jeong et al., 2013). The study was subsequently validated in other pre-clinical models of OA (Barry and Murphy, 2013). In general, this phenomenon transfers to other disease scenarios, as reviewed comprehensively by Prockop et al. (Prockop, 2009). More recently, phase I trials have provided evidence that MSCs also have clinical utility in modulating OA (Jo et al., 2014; Pers et al., 2016); a number of unpublished Phase 2 trials are ongoing assessing adipose-derived MSCs in OA including ADIPOA2 (http://adipoa2.eu/).

**Figure 1:**
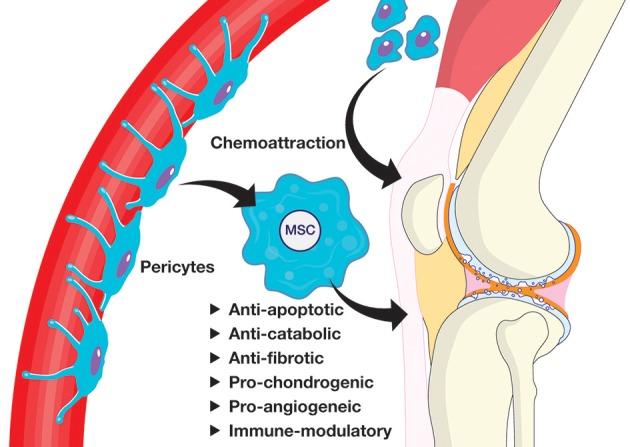
Proposed mechanism of action for tissue repair by endogenous MSCs.

MSCs disappear from the target tissue quickly after administration, but are still able to deliver chondroprotective and immunomodulatory effects (ter Huurne et al., 2012). Since their therapeutic efficacy seems to be independent of their engraftment, it is now considered to be mainly paracrine-mediated. The increasingly accepted model is that MSCs are found dormant *in vivo* as pericytes (Crisan et al., 2008). These participate in the development of tissues, including synovium, and are involved in tissue repair during adult life (Roelofs et al., 2017). Once activated in response to signals associated with the injured environment, such as pro-inflammatory cytokines, a phenomenon generally referred to as “licensing,” they secrete factors, including chemokines and cytokines, to establish a regenerative environment. Depending on the environment of the specific disease, anti-apoptotic and anti-fibrotic factors may limit the extent of damage to improve tissue healing (Ryan et al., 2017). Tissue-intrinsic progenitors are prompted to proliferate and differentiate, while chemoattractants recruit endogenous progenitors to the site of injury. Concurrently, activated MSCs are capable of modulating the immune response locally by selectively inhibiting the proliferation of immune cells (Aggarwal and Pittenger, 2005) (Figure 1). This paper will review the evidence for these therapeutic effects in models relevant to OA, either *in vivo* or *in vitro* (summarized in Table 1). It will be critical in the future to validate those findings using freshly isolated stromal cells.

**Table 1:** The MSC secretome and OA/cartilage protection.

**Activity**	**Factor**	**References**
Anti-apoptosis	STC-1,	Rehman et al., 2004; Block et al., 2009
Anti-fibrosis	bFGF, AMD, HGF	Li et al., 2009; Suga et al., 2009; Maumus et al., 2013
Tissue metabolism	TIMP-1, TIMP-2	Lozito and Tuan, 2011
Chondrogenesis	TSP2	Jeong et al., 2013, 2015
Immunosuppression	PGE2	Aggarwal and Pittenger, 2005; Sotiropoulou et al., 2006; Martinet et al., 2009
Immunosuppression	TSG-6	Mindrescu et al., 2000; Bárdos et al., 2001; Lee et al., 2009
Anti-apoptosis	ECVs	Liu et al., 2018a,b
Immunosuppression	ECVs	Mokarizadeh et al., 2012; Budoni et al., 2013; Zhang et al., 2014
Chondrogenesis	ECVs	Zhu et al., 2017
Chondroprotective/ anti-inflammatory effects	ECVs	Cosenza et al., 2017

## The MSC Secretome

MSCs display a rich secretory profile which is enhanced by exposure to inflammatory signals. A proteomics approach identified 118 proteins differentially expressed by human adipose-derived stem cells (ASCs) upon tumor necrosis factor (TNF)-α stimulation (Lee et al., 2010). These included many cytokines and chemokines [interleukin (IL)-6, 8; chemokine (C-X-C motif) ligand or CXCL2, 5, 6, and 10 and monocyte chemoattractant protein 1 (MCP1)], proteases and protease inhibitors [matrix metalloproteinases (MMP)-1 and 2, tissue inhibitors of metalloproteinases (TIMP)-1 and 2], extracellular matrix (ECM) molecules and factors involved in immune regulation and cell signaling.

### Apoptosis

Chondrocyte apoptosis has been associated with degenerative OA for many years (Aigner et al., 2004; Del Carlo and Loeser, 2008). Although there are no reports of direct anti-apoptotic effects of MSCs in the context of OA, indirect evidence suggests that exosomes obtained from human MSCs, and by inference comprised of secreted factors, inhibited IL-1β-induced apoptosis of *ex vivo*-cultured OA chondrocytes (Liu et al., 2018a). Additionally, MSC-derived exosomes promoted chondrocyte proliferation in a rat model of OA, by blocking miR-206 with lncRNA-KLF3-AS1 (Liu et al., 2018b). Despite soluble factors were not shown in models of OA, MSCs responded to two different apoptotic cell lines *in vitro* by increased expression and secretion of the anti-apoptotic hormone stanniocalcin (STC)-1 (Block et al., 2009). Future work looking at joint-associated MSC anti-apoptotic effects is likely to identify direct mediators of the process.

### Fibrosis

Maumus et al. co-cultured autologous ASCs with chondrocytes derived from OA patients in a transwell system (Maumus et al., 2013). The authors observed marked decreases in expression levels of hypertrophic and fibrotic markers MMP-13, alkaline phosphatase, Runx2, collagens type I, III, VI and vimentin, as well as a 40% increase in TGF-β1 secretion. By using a neutralizing antibody, HGF was identified as the main mediator of the anti-fibrotic effect. This data is of particular relevance as HGF concentration in synovial fluid has a direct correlation with the severity of OA (Dankbar et al., 2007). MSCs also inhibit fibrosis *in vivo* through bFGF (Suga et al., 2009) and adrenomedullin (Li et al., 2009). In addition, a number of studies proposed that *in vivo*-administered MSCs secreted TSG-6 and indirectly prevented fibrosis by suppressing the early inflammatory response to various diseases other than OA, including myocardial infarction (Lee et al., 2009), peritonitis (Choi et al., 2011), inflammation of the cornea (Oh et al., 2010), and cornea allograft rejection (Oh et al., 2012).

### Tissue Metabolism

Amongst other activities, MMPs break down ECM and are regulated by specific inhibitors called TIMPs. In OA, the balance between anabolic and catabolic factors is disrupted in favor of the latter (Lohmander et al., 1993). MMP-2,-9, and -13 were detected at higher levels in human OA cartilage compared to healthy tissue (Jackson et al., 2014). Furthermore, decreased MMP-13 correlated with improved osteochondral repair in rats treated with doxycycline (Lee H. et al., 2013) and MSCs constitutively secrete high levels of TIMP-2 and -1, which inhibit MMP-2 and MMP-9, respectively. Under pathological stress (IL-1β, TNF-α, hypoxia) TIMP-1 secretion is upregulated to counteract increased catabolic activity (Lozito and Tuan, 2011). In addition, MMP inhibition is not specific; TIMP-1 can inhibit most MMPs (Visse and Nagase, 2003), making MSCs an even more versatile tool for restoring the metabolic balance of degenerating cartilage.

### Chondrogenesis

Matricellular proteins, secreted matrix proteins with regulatory roles, bind to ECM and act as receptors for cell-surface molecules, growth factors and MMPs (Bornstein et al., 2000). Thrombospondin (TSP2) for example is a known regulator of cartilage and bone differentiation and is secreted by MSCs to induce proliferation via autocrine mechanisms (Hankenson and Bornstein, 2002). TSP2, secreted by human umbilical cord blood-derived (UCB)-MSCs treated with synovial fluid from OA patients, induced differentiation of chondroprogenitor cells. It promoted cartilage regeneration in a rabbit full-thickness osteochondral-defect model (Jeong et al., 2013). TSP2 was found to have an autocrine action on human UCB-MSCs, BM-MSCs and ASCs, promoting cartilage differentiation and preventing hypertrophy (Jeong et al., 2015). Although data is limited, there is evidence that TSP2 is one of the main paracrine players in MSC-mediated cartilage regeneration.

### Immunosuppression

The role of inflammation in the establishment and maintenance of OA is now widely accepted (Ayral et al., 2005) with synovial membrane inflammation a hallmark of OA pathology (Goldring, 1999; Pelletier et al., 2001). Histological studies show that OA patients have variable degrees of synovitis, with higher levels of pro-inflammatory cytokines and infiltration of immune cells, predominantly macrophages (Benito et al., 2005). Biological markers of inflammation positively correlate with knee pain (Baker et al., 2010; Scanzello et al., 2011) and clinical progression of the disease (Krasnokutsky et al., 2011; Roemer et al., 2011). Licensed MSCs secrete an array of anti-inflammatory cytokines which can help re-establish an equilibrium in the inflamed synovium: MSC-conditioned medium (CM) decreased production of inflammatory mediators in OA joint explants (van Buul et al., 2012).

Di Nicola et al. first assessed the potential for allogeneic MSC rejection in a mixed lymphocyte reaction (MLR). Instead of evoking an immune response, the cells suppressed proliferation of T-cells (Di Nicola et al., 2002; Krampera et al., 2003). The relevance of MSC-secreted factors in immunomodulation was shown by the capacity of their supernatant to divert immune cells from injured organs (Parekkadan et al., 2007). Currently, MSCs are under evaluation in numerous clinical trials for many inflammatory conditions (Trounson and McDonald, 2015).

One of the main effectors of MSC-mediated immune-suppression is prostaglandin-E2 (PGE2). PGE2 is constitutively secreted by MSCs and its production is dramatically enhanced via stimulation by interferon (IFN)-γ, TNF-α (English et al., 2007), or IL-1β (Chen et al., 2010). PGE2 negatively affects the proliferation of T- (Martinet et al., 2009) and natural killer (NK) cells (Sotiropoulou et al., 2006), causes an increase in the pool of regulatory T (Treg) cells, stimulates macrophages to produce IL-10 and prevents monocytes from differentiating into dendritic cells (DCs) (Aggarwal and Pittenger, 2005). In OA, PGE2 mediates ASC therapeutic effects and is a regulatory checkpoint in immune-modulation. Manfredini et al. provided evidence that the PGE2/COX2 pathway is responsible for the induction of IL-10 and inhibition of TNFα and IL-6 to induce an M2 switch in human synovial macrophages (Manferdini et al., 2017).

Indoleamine 2,3-dioxygenase (IDO) catalyzes the breakdown of tryptophan, causing suppression of T-cells. It is employed by DCs to modulate immune responses (Mellor and Munn, 2004), but can be secreted by MSCs upon IFN-γ stimulation (Krampera et al., 2006). In a human MLR, IFN-γ-induced expression of IDO in MSCs was responsible for suppression of T-cell proliferation (Meisel et al., 2004). It also drives M2 polarization in macrophages and induces a tolerogenic phenotype in DCs and Tregs (Ge et al., 2010; Sica and Mantovani, 2012). Its' importance in MSC-mediated immunosuppression has been validated using specific inhibitors and knockout MSCs (Krampera et al., 2006; English et al., 2007; Spaggiari et al., 2008).

TNF-inducible gene (TSG)-6 is known for its multiple and diverse anti-inflammatory mechanisms (Wisniewski and Vilcek, 2004). Produced in response to inflammatory signals, it has a pivotal role in MSC-mediated immunosuppression (Lee et al., 2009). On the other hand, it was identified as one of the most significantly up-regulated genes in human OA articular cartilage (Chou et al., 2015) and proposed as a disease biomarker, as its activity in synovial fluid predicted OA progression (Wisniewski et al., 2014). TSG-6 has a complex role in cartilage pathology, as it is involved in matrix assembly during synthesis of new tissue (Chou et al., 2018).

Other molecules have been shown to mediate MSC immunosuppression, such as C-C motif ligand 2 (CCL2) (Rafei et al., 2009), galectins (Sioud et al., 2011), IL-6 (Scheller et al., 2011), and TGF-β (Di Nicola et al., 2002). None of these factors has an exclusive role; their functions may be redundant and/or synergistic. To fully express an anti-inflammatory phenotype, MSCs need to be licensed. This can be achieved in response to IFN-γ alone (Krampera et al., 2006) or in combination with TNF-α, IL-1α, or IL-1β (Ren et al., 2008). Additionally, IL-1β, granulocyte-colony stimulating factor (G-CSF), stromal cell-derived factor 1 (SDF1) and stem cell factor (SCF) induced differential expression of numerous cytokines in MSCs after only 2 h of treatment (Czekanska et al., 2014). Licensed MSCs have an improved regenerative capacity in pre-clinical models, with better homing potential (Duijvestein et al., 2011) and recruitment of host immune cells (Lee S. et al., 2013).

### The Role of Apoptotic MSCs

Once administered, MSCs can undergo biological changes more radical than differentiation or licensing. Toupet et al. observed that most MSCs disappear 10 days post-IA injection in a murine model of OA, with similar results obtained with syngeneic and xenogenic human ASCs (Toupet et al., 2013, 2015). Despite death and clearance of administered cells, significant therapeutic effects are observed in response to IA injection of mouse ASCs (ter Huurne et al., 2012; Schelbergen et al., 2014).

**Figure 2:**
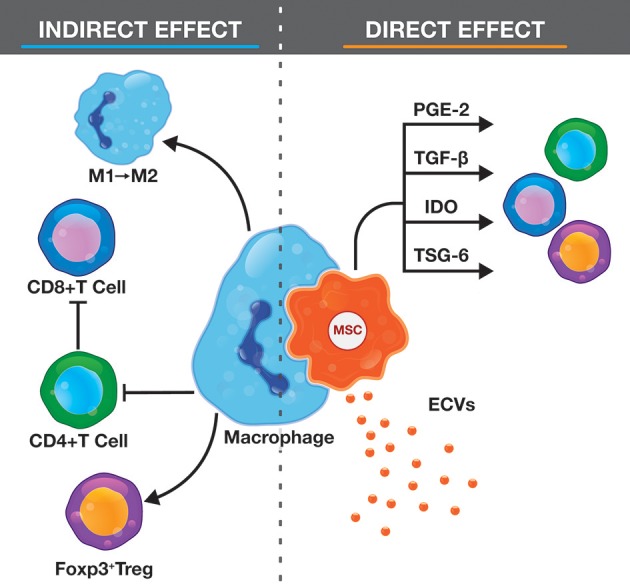
Representation of the immunomodulatory effects of apoptotic MSCs.

Apoptotic cells communicate with immune cells through two different mechanisms: direct effects associated with apoptotic cells themselves and indirect effects triggered in phagocytizing cells (Figure 2). Direct effects include secretion of IL-10 and TGF-β, generating an immunosuppressive microenvironment (Chen et al., 2001; Korns et al., 2011). This milieu inhibits lipopolysaccharide (LPS)-stimulated macrophages from secreting IL-1β and TNF-α (McDonald et al., 1999). Indirect effects are associated with elimination of apoptotic cells by phagocytes, resulting in reduced responsiveness to LPS (Perruche et al., 2009) and a switch to an anti-inflammatory profile (Fadok et al., 1998). Immune cells that internalize apoptotic cells also fail to induce CD4^+^ T helper cells, leaving the effector lymphocytes in a “helpless” state (Griffith et al., 2007) and induce clonal expansion of Foxp3^+^ Treg cells (Xia et al., 2007; Perruche et al., 2008).

Using a murine model of graft-vs.-host disease (GvHD), researchers demonstrated that infused MSC apoptosis is induced by recipient T cells through cell-to-cell contact with release of perforin- and granzyme B-containing granules (Galleu et al., 2017). Phagocytes were also shown to have a key role producing IDO upon engulfing apoptotic MSCs. When these components were knocked down or inhibited, the therapeutic efficacy of MSCs was lost. Most importantly, infusion of MSCs rendered apoptotic *ex vivo* restored therapeutic effects. Interestingly, patient responsiveness to MSCs correlated with their cytotoxic capacity. These findings provide evidence that apoptosis is one of the driving mechanism of MSC-mediated immunosuppression.

TGF-β-mediated tolerance induction is the most commonly reported mechanism in pre-clinical studies of extracorporeal photopheresis, the administration of leukocytes rendered apoptotic *ex vivo*. A strong immunomodulatory effect was observed in inflammatory arthritis (Michlewska et al., 2009; Perruche et al., 2009) and photopheresis is an approved therapy for cutaneous T cell lymphoma and GvHD (Weitz et al., 2015). Apoptosis may also represent an important component of MSC therapy in OA. Unpublished data in our laboratory shows as low as 1.6% MSC engraftment 3 days after IA administration of GFP^+^ MSCs in murine OA knees. Fluorescent cells were not detected in any adjacent tissue, including local lymph nodes. This reinforces the hypothesis that implanted cells could undergo apoptosis and modulate inflammation with subsequent protection from OA development. Whereas, apoptosis post-infusion is a transient event, Galleu et al. showed that the subsequent response might represent a reprogramming of certain aspects the host immune system (Galleu et al., 2017).

## Looking Further: Extra-Cellular Vesicles

The paracrine action of MSCs is not limited to soluble factors. MSCs, like many other cells, have been shown to produce extracellular vesicles (ECVs) (Lai et al., 2010), small structures enclosed in a phospholipid bilayer, carrying many cytoplasmic components. ECVs are involved in intercellular communication through horizontal transfer of mRNA and protein and are grouped based on size, with different composition and biogenesis. Exosomes range between 40 and 100 nm in diameter. They are constitutively released from the late endosomal compartment by fusion of multivesicular bodies with the plasma membrane, but their production can increase upon cytoskeleton activation. Exosomes are characterized by proteins required for their formation and transport, such as tetraspanins, Alix and tumor susceptibility gene 101. Microvesicles are a heterogeneous population of ECVs between 100 and 1,000 nm generated via direct budding upon activation by a stress signal, which alters the phospholipid balance of the membrane, forming lipid rafts. Microvesicles are characterized by membrane markers specific to the parent cell type. In pre-clinical models, ECVs were observed to have anti-apoptotic (Bruno et al., 2012), anti-fibrotic (Li et al., 2013), pro-angiogenic (Bian et al., 2014), and anti-inflammatory effects (Lee et al., 2012). MSC-derived ECVs induce generation of Tregs, inhibit proliferation of lymphocytes (Mokarizadeh et al., 2012), macrophages (Zhang et al., 2014), and B cells (Budoni et al., 2013). However, ECVs alone may fail to deliver the same immunomodulatory effects of parental cells, with cell-cell contact still required to modulate lymphocyte proliferation and function (Conforti et al., 2014).

MSC-derived ECVs produced promising results in rat models of osteoporosis (Qi et al., 2016) and osteochondral defect repair (Zhang et al., 2016). More recently, MSC-ECVs were tested in OA models. Exosomes derived from synovium MSCs and induced pluripotent stem cells attenuated disease scores in a collagenase-induced OA (CIOA) mouse model, by promoting chondrocyte proliferation and migration (Zhu et al., 2017). Notably, exosomes derived from synovial MSCs overexpressing miR-140-5p induced proliferation of chondrocytes *in vitro*. When administered in a rat model of OA disease progression and cartilage degeneration were significantly delayed (Tao et al., 2017). Cosenza et al. delivered MSC-ECVs in a CIOA model and reported reduced joint damage (Cosenza et al., 2017). The use of MSC-ECVs as a therapy for OA would bring many advantages compared to cell-derived products, avoiding concerns of possible malignant transformations. However, issues may arise with ECV production as they may need to be specifically tailored for the indication to be treated. Additionally, their manufacture is not as yet standardized for clinical production, as is the case for cellular products.

## Conclusions

Reports summarized here suggest significant potential for the use of MSCs or MSC-CM in OA. *In vitro*, co-culture of OA chondrocytes with ASC-CM resulted in NF-κB-mediated cytoprotective effects via enhanced production of collagen II, inhibition of IL-6, TNF and various MMPs, as well as upregulation of IL-10 (Platas et al., 2013). Similarly, using OA cartilage explants, MSC-CM was shown to interfere with the NF-κB pathway to mediate anti-inflammatory and anti-catabolic effects (van Buul et al., 2012). MSCs have already proved to be a valuable tool for many conditions, including acute GvHD (Le Blanc et al., 2008) and multiple sclerosis (Karussis et al., 2008).

Phase I clinical trials have demonstrated the safety of direct IA administration of MSCs in OA patients (Centeno et al., 2008; Davatchi et al., 2011). In 2012, pain reduction was reported up to 6 months after injection of 20–24 million MSCs, with increased cartilage thickness and reduction of edematous subchondral patches in three out of six patients (Emadedin et al., 2012). Jo et al. injected higher doses of ASCs (up to 10 × 10^8^), obtaining significantly improved WOMAC score with a clinically meaningful pain reduction and, most importantly, regenerated hyaline articular cartilage in the most severely degenerated site in the knee (Jo et al., 2014). In the ADIPOA trial, a single dose of 2 million ASCs significantly improved pain levels and function (Pers et al., 2016).

In summary, MSCs may act through a hit-and-run mechanism rather than stably engrafting in the tissue. Autopsies of patients that received MSC IV infusions for different conditions within a year before death confirm that donor MSCs are not normally retained in the host tissue. Detection of donor DNA did not correlate with the degree of HLA mismatch or the clinical response, suggesting that clearance is not immune-mediated (von Bahr et al., 2012). However, the role of cell death in mediating the therapeutic effects of MSCs needs further investigation and the phenotype and activity of cells that survive even for a short time at the site of implantation elucidated.

## Author Contributions

PM: contributed to the development of the concept underpinning the review, researched the relevant literature, and wrote the body of the review; SR: also contributed to the concept of the submission and helped with the writing; AG: researched the literature about apoptosis; JMM: participated in manuscript preparation, on-going and final review of the submission; FB: also participated in manuscript conception and review.

### Conflict of Interest Statement

The authors declare that the research was conducted in the absence of any commercial or financial relationships that could be construed as a potential conflict of interest.
